# Assessment of Opioid Prescribing Practices Before and After Implementation of a Health System Intervention to Reduce Opioid Overprescribing

**DOI:** 10.1001/jamanetworkopen.2018.2908

**Published:** 2018-09-28

**Authors:** Barry R. Meisenberg, Jennifer Grover, Colson Campbell, Daniel Korpon

**Affiliations:** 1 Center for Health Care Improvement, Department of Medicine, Anne Arundel Health System, Annapolis, Maryland; 2 Johns Hopkins School of Public Health, Baltimore, Maryland; 3 Department of Informatics, Anne Arundel Health System, Annapolis, Maryland

## Abstract

**Importance:**

Overprescribing of opioids has generated and sustains the opioid overdose epidemic. Health systems have a responsibility to lead the effort to reduce overprescribing.

**Objective:**

To measure the effects of multilevel interventions on opioid prescribing within a health system.

**Design, Setting, and Participants:**

Quality improvement study comparing a 6-month preintervention baseline with a 16-month postintervention period ending in April 2018. Inpatient and outpatient clinical activity within a regional health system including an acute care hospital, same-day surgery, and outpatient clinics. Opioid prescribing activity by hundreds of clinicians involving over a million clinical encounters was measured using a health system’s electronic medical record.

**Interventions:**

Multiple parallel interventions in different domains, including prescriber education and accountability, enhanced oversight via measurement of individual prescribers, tools to right-size postoperative discharge prescriptions, reduction of default amounts on standard opioid prescription orders, and professionally written patient and public education about opioid risks and alternatives.

**Main Outcomes and Measures:**

Morphine milligram equivalents (MME) per encounter per month, MME per opioid prescription, and rate of opioid prescriptions (opioid prescriptions per encounter per month).

**Results:**

More than 44 000 clinical encounters per month were recorded. All baseline trends were not significantly different from 0. Total health system MME per encounter decreased 1.0 MME per encounter per month. At the end of the postintervention observation period, the monthly MME per encounter was 58% lower than the average of the 6-month baseline, the MME per opioid prescription per month was 34% less than the average of the baseline, and the opioid prescription rate was 38% lower than the average of the baseline.

**Conclusions and Relevance:**

Opioid overprescribing was reduced with multifocal interventions targeting patient and public demand, creating prescriber awareness and accountability, and creating tools for clinical leadership accountability. The interventions described are adoptable by most organized health systems. Reducing total opioid supply within communities should be given high priority by those with a mission to protect and improve public health.

## Key Points

**Question:**

Can a series of focused interventions targeting different aspects of opioid overprescribing reduce opioid overprescribing within a health system?

**Findings:**

This quality improvement study found postintervention reductions in opioid prescribing occurred at a statistically significantly faster pace than a preintervention downward trend. Overall reductions for key measures were 58% for morphine milligram equivalents per clinical encounter per month, 34% for morphine milligram equivalents per opioid prescription, and 38% for opioid prescriptions per encounter.

**Meaning:**

Overprescribing can be reduced in different clinical departments using tools available to most health systems.

## Teaser

This quality improvement study compares opioid prescribing rates in a single health system before and after a multipart intervention intended to decrease overprescribing.

## Introduction

The rapidly increasing death rate in the United States from opioid overdoses has attracted national and international attention in both clinical circles and in the public space.^1^ States of emergency have been declared both at the federal level and in local jurisdictions. A diverse array of solutions have been proposed, including criminal justice procedures, health policies, funding allocations, and reforms in clinical practice.^2,3,4^ The rapid growth in opioid overdose deaths, mainly from so-called street drugs such as heroin, fentanyl, and fentanyl derivatives, was preceded by a 300% expansion of retail opioid prescribing beginning in the early 1990s and peaking in 2012.^5^ But even with a slight decline since 2012, prescribing far exceeds 1991 levels and that of other nations.^6^ The causes of this massive prescribing growth have been much debated.^7,8,9^ But it is not debatable that prescribed opioids, including diverted prescription opioids, were the initial source of opioids for most current heroin users.^10^ The prescriber’s role in generating and sustaining opioid abuse has been made clear by studies that link a practitioner’s prescribing patterns to a patient’s likelihood of long-term opioid dependence.^11,12^ This linkage between prescribing patterns and opioid dependency^7,8,9,10,11,12^ formed the rationale for this targeted initiative to reduce opioid prescribing locally.

In response to the local public health emergency and declared state of emergency, the authors initiated a pilot review of prescribing among physicians, physician assistants, and nurse practitioners in primary care and emergency departments (EDs) using the health system’s electronic medical record (EMR) to collect data on prescribing frequency and overall opioid volume. The pilot analyses revealed high variability in prescribing among practitioners and other deficiencies in management of chronic opioid users, providing the rationale for a comprehensive campaign. The specific aims were to reduce unwarranted variability in opioid prescribing; standardize prescribing for clinically similar situations; reduce total opioid prescribing; create accountability from prescribers and medical directors by collecting and sharing prescribing data from the EMR; and observe for adverse effects by monitoring patient satisfaction with pain management and return visits to the ED. This report describes the initiatives undertaken and the results of a physician-led campaign to reduce opioid prescribing within a health system.

## Methods

This report conforms to the Standards for Quality Improvement Reporting Excellence (SQUIRE) reporting guideline for health care safety innovations. The Clinical Research Committee of Anne Arundel Health System reviewed this project and determined that it qualified as quality improvement and not research. It also performed an ethics review and did not identify any concerns in initiating the interventions.

Anne Arundel Medical Center is a 385-bed acute care hospital in Anne Arundel County, Maryland, serving as a regional referral center for more than 1 000 000 individuals. Relevant volumes for 2017 include 25 567 medical admissions, excluding newborns, 96 118 visits to the ED, 17 791 inpatient surgical procedures, 6871 ambulatory surgical procedures, and 331 554 000 visits to ambulatory health system clinicians. The health system directly employs more than 326 physicians, nurse practitioners, and physician assistants, including 42 primary care practitioners in 15 separate locations. All clinical activity uses the health system EMR Epic 2015.

### Prescribing Practices

A standing report was created that identified all prescription opioids and converted the prescription dose into morphine milligram equivalents (MME) using a standard reference table.^13^ The prescription was then assigned to individual prescribers and organized by particular clinical department or service: primary care, ED, acute care hospitalist, medical specialists, orthopedic clinic, oncology, palliative care, and surgery. Opioid prescriptions from oncology and palliative care were excluded from the analysis as public policies did not seek to change opioid prescribing for patients with advanced cancer or patients in palliative care for painful conditions. The health system employs no pain management specialty physicians. A few iterations were required until prescribers were accurately assigned to specific clinical groups. Individual service reports, which updated daily, were built for medical directors allowing comparison among prescribers. The reports included patient medical record numbers so that responsible medical directors could review the medical record to determine the clinical circumstances of the prescription.

Prescribing trends were overseen by a newly created Opioid Prescribing Task Force composed of clinical and administrative leaders from pharmacy, mental health, surgery, orthopedics, primary care, ED, community health, informatics, quality, and health system administration.

A multilevel campaign was initiated comprising large group education that included departmental grand rounds, service meetings with data review, and circulation of medical journal articles with information on overprescribing. A visual display of individual clinician prescribing for ready comparison with service peers was created. This was followed by academic detailing^14^ by medical directors with one-on-one meetings with prescribers to reinforce the key points of the education and review individual prescribing data and comparison with peers. The topics and main points of the education efforts are shown in eTable 1 in the Supplement. The principal goal was to convey an appreciation for the idea that medicinal opioids generated and sustain the opioid overuse epidemic and that prescriber behavior influences patient outcomes with regard to opioid dependence.

Although all members of the medical staff were included in the education efforts and had access to all tools introduced, 3 clinical areas, the ED, primary care, and the orthopedic clinic, were chosen for initial focused efforts because of high opioid prescription rate and volume. Some clinical departments, such as the ED and upper extremity orthopedic surgeons, rapidly adopted service-specific standardized approaches to prescribing following guidance to use the smallest amounts of opioid that might still be effective.^4^ Other departments, such as obstetrics and surgery, created these standardized approaches during the course of the intervention year.

New EMR-based tools included patient education on opioid alternatives and opioid safety, identification of patient-level characteristics associated with elevated risk for dependence,^15^ facilitated referral for substance abuse counseling, and detoxification within the EMR. An easy-to-use tracking tool for opioid consumption during an inpatient stay was created to help reduce mismatched patient need and discharge prescription amounts, a problem that has been cited as a cause of overprescribing.^16^

### General Public Education

The goal was to enhance the public’s level of caution about opioids and thereby reduce demand for unnecessary opioids in any potential medical encounter. Professionally developed written materials from the Anne Arundel County Department of Health in English and Spanish were reviewed with a cadre of patient advisors. The materials were used to communicate with the public about risks of opioid use, alternatives to opioids, safe use, safe storage, and disposal. Messages were delivered through various communication channels available to the health system, including local and regional radio and television interviews with physician leaders, authored columns in local newspapers, website articles, social media postings, and signage within the medical center.

### Patient Education

As above, the goal was to enhance the patient’s level of caution about opioids and achieve reasonable pain management goals, particularly after surgery. Similar materials and processes, including use of patient advisors to review materials, were used to develop materials that support the use of nonopioid alternatives for acute or chronic pain. New patient safety instructions were created to accompany every EMR-generated opioid prescription in the after-visit summary, including safe storage and disposal. The instructions included explanation of side effects, including emphasis on risk of sedation and respiratory depression, warnings about concurrent sedating medications and alcohol, safe storage, and disposal of unused medication. Discharge instructions also included the addresses of medication take-back programs. Revised patient education materials regarding appropriate pain management goals were created. eTable 2 in the Supplement shows the time course for initiation of the interventions, all of which were maintained and expanded as interventions disseminated to additional departments, surgical procedures, and services.

### Study of Interventions

The study measured all ambulatory and hospital discharge opioid prescriptions, including same day-surgery, before and after intervention. Patient satisfaction with pain management was measured through an existing survey, the Clinician and Group Consumer Assessment of Healthcare Providers and Systems (CG-CAHPS), to observe for adverse trends in patient satisfaction after the interventions were initiated. Because national data suggest a modest existing trend toward reduction in overall prescribing,^6^ we included a 6-month preintervention data view beginning in April 2016 to assess any secular preintervention trend already under way. Interventions were phased in over a 3-month period beginning in October 1, 2016. The postintervention observation period began on January 1, 2017, and continued through April 30, 2018. To account for any changes in clinical volumes that might affect opioid volume, we collected data on number of encounters using standard health system reports.

### Measures

The volume and rate of opioid prescribing through the EMR were the key outputs measured. Data were analyzed as MME per patient encounter per month, MME per prescription, and number of opioid prescriptions per encounter.

### Statistical Analysis

Separate linear regression analyses were performed for the parameters of aggregated MME per encounter per month, MME per opioid prescription, and rate of opioid prescription per encounter for the 6-month baseline period and for the 16 months of postintervention observation. To avoid contaminating either analysis, the 3 months of intervention phase-in were not included in either data set. All analyses were conducted with Bonferroni confidence interval control of type I error rate (0.05 ÷ 6 = 0.00833). The slopes of the 2 linear regressions, before and after intervention, were calculated. *P* values on the slopes of the preintervention and postintervention trend lines were calculated to determine statistical significance. Two-sided *P* values of less than .05 were considered statistically significant.

## Results

More than 44 000 clinical encounters per month were recorded. The interventions were associated with a reduction in prescribed MME per encounter, MME per prescription, and opioid prescriptions per encounter. All differences in slopes were significant using the Bonferroni confidence interval control method.

**Figure 1.**
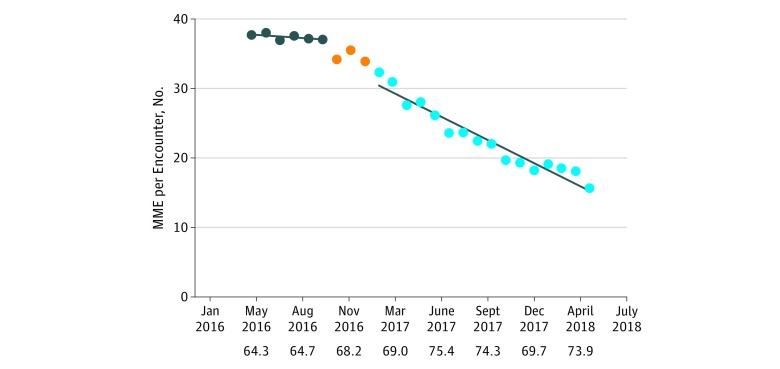
Health System Morphine Milligram Equivalents (MME) per Clinical Encounter Over Time The first 6 data points represent the baseline period, the orange dots represent the 3-month intervention phase-in, and the last 16 data points are the postintervention months. The numbers below the x-axis indicate the average monthly clinical encounters (in thousands) for the preceding quarter.

**Figure 2.**
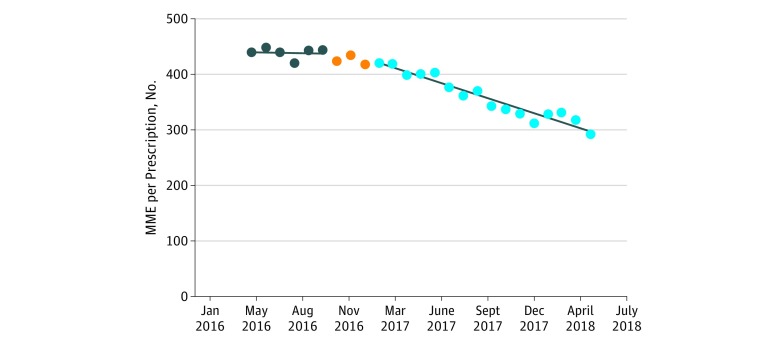
Health System Average Morphine Milligram Equivalents (MME) per Opioid Prescription Over Time The first 6 data points represent the baseline months, the 3 orange dots represent the 3-month intervention phase-in, and the last 16 data points represent the postintervention months.

**Figure 3.**
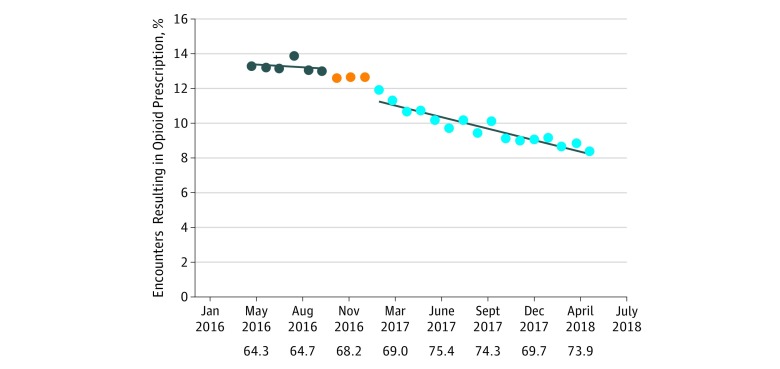
Health System Opioid Prescriptions per Clinical Encounter Over Time The first 6 data points represent the baseline months, the 3 orange dots represent the 3-month intervention phase-in, and the last 16 data points represent the postintervention months. The numbers below the x-axis indicate the average monthly clinical encounters (in thousands) for the preceding quarter.

**Figure 4.**
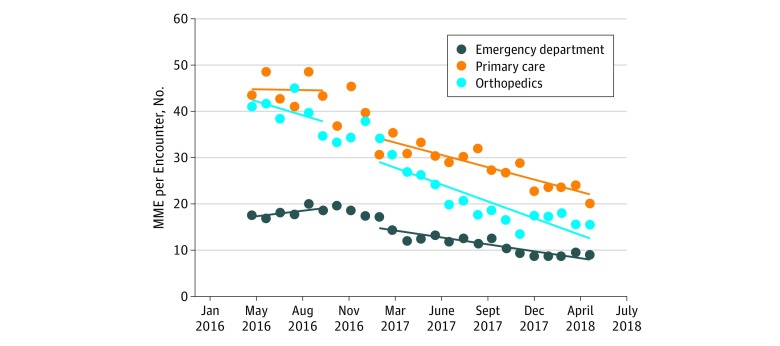
Morphine Milligram Equivalents (MME) per Clinical Encounter Over Time for the 3 Targeted Clinical Areas For each clinical area, the first 6 data points represent the baseline period, the middle 3 dots represent the 3-month intervention phase-in, and the last 16 data points are the postintervention months.

Figure 1, Figure 2, Figure 3, and Figure 4 show prescribing trends for the baseline 6 months and for the 16 months after the interventions. In all cases, the baseline trend was not statistically different from 0 and the postintervention negative slope was highly statistically significant at *P* < .001. Total health system MME per encounter decreased 1.0 MME per encounter per month from a starting level of 34.4 MME per encounter per month (Figure 1). At the end of the postintervention observation period, the monthly MME per encounter was 58% lower than the average of the 6-month baseline. Morphine milligram equivalents per opioid prescription decreased 8 MME per prescription per month from a starting level of 428 MME per prescription per month (Figure 2). At the end of the 16-month postintervention period, the MME per prescription per month was 34% less than the average of the baseline.

The percentage of health system clinical encounters resulting in an opioid prescription was reduced by 0.2% each month from a starting level of 11.5% (Figure 3). At the end of the 16-month postintervention period, the opioid prescription rate was 38% less than the average of the baseline.

Analysis of MME per encounter per month for the 3 initial targeted clinical departments (Figure 4) shows improvement in all 3. Primary care MME per encounter decreased 0.8 from a starting point of 34.8, resulting in a 55% reduction from baseline. Emergency department prescribing decreased 0.4 MME per encounter per month from a starting level of 17. Overall reduction from the average of the baseline 6 months was 49%. Orthopedic clinic MME per encounter decreased 0.9 MME per month from a starting point of 43 MME per encounter. Overall reduction from baseline was 61%.

**Figure 5.**
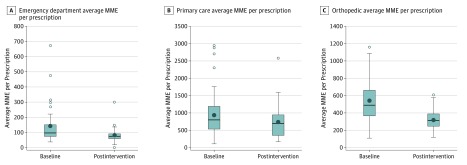
Morphine Milligram Equivalents (MME) per Prescription Plots show the 6-month baseline period and the last 6 months of the postintervention period for each of the 3 targeted clinical areas: emergency department, primary care, and orthopedics. The horizontal lines represent the median. The bottom and top of each box indicate the first and third quartiles, respectively. The points outside the whiskers represent outlying prescribers beyond 1.5 times the interquartile range. The black dots represent the mean.

Variability in MME per encounter was reduced in all 3 targeted areas as measured by the interquartile range (IQR) (Figure 5). In the ED, the IQR decreased from 72 to 29.5 MME per encounter with the number of high outliers decreasing from 5 to 2. Among primary care prescribers, the IQR decreased from 686 to 601 MME per encounter with the number of outliers decreasing from 4 to 0. In the orthopedic clinic, the IQR decreased from 302 to 143 MME per encounter with 1 high outlier in each time period.

Patient satisfaction with pain management in the ED, as measured by the percentage of CG-CAHPS survey respondents who graded their satisfaction as either a 9 or 10 of 10 possible points, did not decrease but rather improved slightly after the interventions from 52% to 61% (eFigure in the Supplement). There was no increase in number of patients who had return visits to the ED within 30 days for pain control after either a surgical procedure or a previous ED visit.

## Discussion

Health systems and their physician leaders have a special responsibility to organize the clinical response to the opioid crisis. Their clinical sites are the source for many opioid prescriptions, they employ or contract with many clinicians both in hospitals and in the community, they are most often the conveners of organized medical staff quality improvement, they have a mission to improve and protect the public’s health, and they have existing channels of communication to educate and inform the public and patients about opioid risks and alternatives.

The success in reducing and standardizing opioid prescribing described in this report is notable for the size and rapidity with which major changes occurred and the large number of clinicians who created the change. Changing physician ordering behaviors on a broad scale is challenging. Successful efforts have been reported with antibiotic use,^17^ laboratory test ordering,^18^ and blood transfusions,^19^ although sustainability of performance improvement is often not demonstrated and there is the potential for submission and publication bias for positive results. Other initiatives, such as efforts to effect cultural change with regard to the ordering of low-value tests, have not succeeded on a broad scale, at least in the early years.^20^

Our data demonstrate that it is possible, through a coordinated, multilevel campaign, to reduce opioid overprescribing without worsening overall patient satisfaction. The intervention was associated with improvements in multiple measures of opioid prescribing: MME per encounter, MME per prescription, percentage of encounters with opioid prescriptions, and variability within 3 targeted departments. The improvements expanded over the observation period as additional departments and procedures initiated prescribing reforms such as postoperative standardization. The success of the current initiative is likely due to the timely union of public and clinician concerns fostered by strong media and government focus on the opioid overdose crisis. In our experience, cautious prescribing has been welcomed by the broad public and engendered little resistance from most patients.

Initial physician concern about the impact of lowered opioid prescribing and patient satisfaction was addressed with both internal and published data showing patient satisfaction with pain management after surgery is not correlated with the volume of prescribed opioids after postsurgical hospital discharge.^21^ Early adopters of conservative prescribing relayed anecdotes of patients expressing gratitude for the discussion about nonopioid alternatives, thus encouraging other clinicians. Another early physician concern regarded the potential loss of prescribing autonomy. But there were no mandated restrictions added into the EMR during the time of this project, although we did add guidance, transparency, internal benchmarking, and accountability. After initial concern about data accuracy, prescribers came to understand that the gravity of the overdose crisis warrants more oversight by clinically informed medical directors. The visual management comparisons were well received, and many expressed surprise to see where they ranked. In some cases, such as the ED and hospitalists, clinicians expressed satisfaction with department-wide standards that made it easier for them to cite adherence to protocols to reduce prescribing volumes.

### Study Strengths

The study had a uniform, reproducible, and validated way of measuring opioid prescribing volume. There was a stable population of prescribers numbering several hundred and the work involved hundreds of thousands of patient encounters. The minimal secular trend in reduced opioid prescribing was measured and accounted for.

### Context

A few published quality improvement projects have shown a reduction in opioid prescribing with efforts to standardize and monitor prescribing patterns involving either a single clinical area^22,23,24,25^ or broadly throughout a health system.^26,27^ In general, these efforts involve adoption of guidelines and monitoring of adherence with data collection. A retrospective 5-year analysis of the impact of standard statewide prescribing guidelines for ED clinicians in Ohio demonstrated a 39.7% decrease in total number of prescriptions and a 28.3% decrease in prescriptions of more than 3 days of opioids.^24^ Within the Veterans Health Administration, an opioid prescribing dashboard was created that allowed facility medical leaders to audit facility-level, prescriber-level, and patient-level prescribing. In the year of follow-up after the advent of the monitoring tool in October 2013, there was an acceleration in the already downward trend in overall opioid prescribing (0.4% per month), high-dose (>100 MME) opioid prescriptions (0.67% per month), and patients receiving concurrent opioids and benzodiazepines (0.86% per month).^26^ A study from Kaiser Permanente Southern California reported the results of a series of interventions rolled out over 6 years that included opioid prescribing data collection and dissemination, dose and duration restrictions, computer decision support meant to discourage long-acting opioids, and warnings against concurrent prescription of benzodiazepines and other high-risk medications.^27^ Improvements were seen in all targeted metrics.

### Limitations

This study was conducted in a single health system with a single EMR that captured all electronic prescriptions. Strong physician leadership helped drive the program. Other health system resources, including marketing and communications, information technology, and medical informatics helped facilitate rapid changes. Other health systems with less overprescribing might see less impressive results on a percentage basis. However, our county is only a moderate prescribing county according to Centers for Disease Control and Prevention data,^28^ so similar gains may be possible in many other jurisdictions. Moreover, the specific tools developed are available to almost any health system or practice group with an EMR.

These results are unlikely to be due to concurrent legislative requirements or other external policies, as the mandatory review of a statewide prescription database had not yet taken effect at the time of these initiatives. Enforced limitations of quantity and preauthorization for high doses among Maryland Medicaid patients did occur halfway through our intervention period, but less than 3% of our encounters are with Medicaid recipients, and these restrictions were modest.^29^

The study lacks prospective controls, as it was not possible to segregate certain clinical areas to avoid the interventions. However, because 6 months of data prior to the interventions showed no statistically significant downward trend, it is likely that the interventions were responsible for the results. Data from the Centers for Disease Control and Prevention showing a much more modest 5.8% reduction in our county, from 74.1 to 69.8 opioid prescriptions per 10 000 population from 2015 to 2016, further support this conclusion.^28^ National data comparing 2017 with 2016 show a reduction of only 12%.^30^

The study is further limited by the fact that, similar to other published reports on opioid prescribing reduction,^22,23,27^ we did not have research access to state or regional prescription drug monitoring programs. Thus, we cannot absolutely exclude the possibility that our significant reduction in internal opioid prescribing resulted from migration of patients to other practitioners not using our EMR. Mass migration out of the health system seems unlikely given the rapidity and scale of the changes noted here and the fact that most of our clinical volumes increased during the intervention year. Finally, our method of studying patient satisfaction with pain management using the CG-CAHPS tool may have failed to detect patient concerns or dissatisfaction among small groups of patients.

Because the solutions were initiated as a bundle, it is impossible to determine which interventions were most effective. The magnitude of the achieved reduction stands out in comparison with other less successful efforts in which either policies alone^31^ or data sharing without follow-up were used.^32,33^ Our program involved 4 key elements, all of which may be necessary for optimum results: patient and family education to reduce demand, achieving prescriber understanding that medicinal opioids are part of the opioid crisis, individual and aggregate data sharing, and oversight by accountable medical directors.

Some observers have noted the trend toward restrictive prescribing and cautioned about too severe a course correction.^34,35^ We share these concerns, but our data did not support the presence of large numbers of dissatisfied patients. Indeed, care was taken to create referral pathways for detoxification programs for those patients interested in addressing long-term habits.

### Future Direction

The process of standardizing prescribing after surgery still needs to be developed fully. While overprescribing is widely acknowledged,^36^ the ideal discharge opioid volume for various procedures remains uncertain. Some have argued that the opioid consumption during the last 24 hours before discharge is the best way to determine prescription volume following discharge.^37^ Indeed, enhanced access to this information was 1 of the tools used in this project. The ability to electronically prescribe opioids remotely through the EMR will facilitate smaller-volume initial prescriptions because a second one can be provided more easily and only if needed.

## Conclusions

Meaningful reductions in opioid overprescribing are feasible and sustainable with central coordination and clinical leadership. The tools described in this report are accessible to most health systems. The drive to standardization for postsurgical prescribing has been facilitated with published reports of the degree of overprescribing and more appropriate postoperative starting doses.^37,38^ We are aware that reducing a community’s opioid reservoir, may, in the short run, increase the number of persons who seek illicit street opioids and thus increase their risk of death. However, we regard cautious prescribing and opioid stewardship as a shared commitment to the long-term health of our community.

## Notes

Supplement. **eTable 1.** Themes of Academic Detailing **eTable 2.** Initiation and Maintenance of Interventions **eFigure**. Patient Satisfaction Graph

## References

[1] Katz J U.S. drug deaths climbing faster than ever. *New York Times* June 6, 2017:A1.

[2] Penm J, MacKinnon NJ, Boone JM, Ciaccia A, McNamee C, Winstanley EL Strategies and policies to address the opioid epidemic: a case study of Ohio. J Am Pharm Assoc (2003). 2017;57(2S):-. doi:10.1016/j.japh.2017.01.001 28189539 PMC5497298

[3] Gostin LO, Hodge JG Jr, Noe SA Reframing the opioid epidemic as a national emergency. JAMA. 2017;318(16):1539-1540. doi:10.1001/jama.2017.13358 28832871

[4] Dowell D, Haegerich TM, Chou R CDC guideline for prescribing opioids for chronic pain—United States, 2016. JAMA. 2016;315(15):1624-1645. doi:10.1001/jama.2016.1464 26977696 PMC6390846

[5] Guy GP Jr, Zhang K, Bohm MK, Vital Signs: changes in opioid prescribing in the United States, 2006-2015. MMWR Morb Mortal Wkly Rep. 2017;66(26):697-704. doi:10.15585/mmwr.mm6626a4 28683056 PMC5726238

[6] Humphreys K Americans use far more opioids than anyone else in the world. *Washington Post* March 15, 2007 https://www.washingtonpost.com/news/wonk/wp/2017/03/15/americans-use-far-more-opioids-than-anyone-else-in-the-world/?utm_term=.1775741df58a. Accessed August 6, 2018.

[7] Baker DW History of The Joint Commission’s pain standards: lessons for today’s prescription opioid epidemic. JAMA. 2017;317(11):1117-1118. doi:10.1001/jama.2017.0935 28241189

[8] Chhabra N, Leikin JB The Joint Commission and the opioid epidemic. JAMA. 2017;318(1):91-92. doi:10.1001/jama.2017.6694 28672310

[9] King NB, Strumpf E, Harper S Has the increase in disability insurance participation contributed to increased opioid-related mortality? Ann Intern Med. 2016;165(10):729-730. doi:10.7326/M16-0918 27571480

[10] Compton WM, Jones CM, Baldwin GT Relationship between nonmedical prescription-opioid use and heroin use. N Engl J Med. 2016;374(2):154-163. doi:10.1056/NEJMra1508490 26760086 PMC11784537

[11] Shah A, Hayes CJ, Martin BC Characteristics of initial prescription episodes and likelihood of long-term opioid use—United States, 2006-2015. MMWR Morb Mortal Wkly Rep. 2017;66(10):265-269. doi:10.15585/mmwr.mm6610a1 28301454 PMC5657867

[12] Barnett ML, Olenski AR, Jena AB Opioid-prescribing patterns of emergency physicians and risk of long-term use. N Engl J Med. 2017;376(7):663-673. doi:10.1056/NEJMsa1610524 28199807 PMC5428548

[13] Centers for Medicare & Medicaid Services. Opioid morphine equivalent conversion factor. https://www.cms.gov/Medicare/Prescription-Drug-Coverage/PrescriptionDrugCovContra/Downloads/Opioid-Morphine-EQ-Conversion-Factors-March-2015.pdf. Accessed December 3, 2017.

[14] Alosa Health Foundation. Academic detailing programs. http://alosahealth.org/our-solutions/academic-detailing-programs. Accessed December 3, 2017.

[15] Bauer SR, Hitchner L, Harrison H, Gerstenberger J, Steiger S Predictors of higher-risk chronic opioid prescriptions in an academic primary care setting. Subst Abus. 2016;37(1):110-117. doi:10.1080/08897077.2015.1129020 26848633

[16] Chen EY, Marcantonio A, Tornetta P III Correlation between 24-hour predischarge opioid use and amount of opioids prescribed at hospital discharge. JAMA Surg. 2018;153(2):e174859. doi:10.1001/jamasurg.2017.4859 29238810 PMC5838710

[17] Sharp AL, Hu YR, Shen E, Improving antibiotic stewardship: a stepped-wedge cluster randomized trial. Am J Manag Care. 2017;23(11):e360-e365. 29182356

[18] Feldman LS, Shihab HM, Thiemann D, Impact of providing fee data on laboratory test ordering: a controlled clinical trial. JAMA Intern Med. 2013;173(10):903-908. doi:10.1001/jamainternmed.2013.232 23588900

[19] Goodnough LT, Shah N The next chapter in patient blood management: real-time clinical decision support. Am J Clin Pathol. 2014;142(6):741-747. doi:10.1309/AJCP4W5CCFOZUJFU 25389326

[20] Rosenberg A, Agiro A, Gottlieb M, Early trends among seven recommendations from the Choosing Wisely Campaign. JAMA Intern Med. 2015;175(12):1913-1920. doi:10.1001/jamainternmed.2015.5441 26457643

[21] Lee JS, Hu HM, Brummett CM, Postoperative opioid prescribing and the pain scores on Hospital Consumer Assessment of Healthcare Providers and Systems Survey. JAMA. 2017;317(19):2013-2015. doi:10.1001/jama.2017.2827 28510669 PMC5815008

[22] Chen JH, Hom J, Richman I, Asch SM, Podchiyska T, Johansen NA Effect of opioid prescribing guidelines in primary care. Medicine (Baltimore). 2016;95(35):e4760. doi:10.1097/MD.0000000000004760 27583928 PMC5008612

[23] Osborn SR, Yu J, Williams B, Vasilyadis M, Blackmore CC Changes in provider prescribing patterns after implementation of an emergency department prescription opioid policy. J Emerg Med. 2017;52(4):538-546. doi:10.1016/j.jemermed.2016.07.120 28111065

[24] Weiner SG, Baker O, Poon SJ, The effect of opioid prescribing guidelines on prescriptions by emergency physicians in Ohio. Ann Emerg Med. 2017;70(6):799-808.e1. doi:10.1016/j.annemergmed.2017.03.057 28549620

[25] Urton MS, Rohlik E, Farrell M, Ng W, Woodard EK Decreasing opioid utilization in rehabilitation patients using a clinical nurse specialist pain consultant program. Arch Phys Med Rehabil. 2017;98(12):2491-2497. doi:10.1016/j.apmr.2017.05.026 28668543

[26] Lin LA, Bohnert ASB, Kerns RD, Clay MA, Ganoczy D, Ilgen MA Impact of the Opioid Safety Initiative on opioid-related prescribing in veterans. Pain. 2017;158(5):833-839. doi:10.1097/j.pain.0000000000000837 28240996

[27] Losby JL, Hyatt JD, Kanter MH, Baldwin G, Matsuoka D Safer and more appropriate opioid prescribing: a large healthcare system’s comprehensive approach. J Eval Clin Pract. 2017;23(6):1173-1179. doi:10.1111/jep.12756 28707421

[28] Centers for Disease Control and Prevention U.S. county prescribing rates, 2016 https://www.cdc.gov/drugoverdose/maps/rxcounty2016.html. Accessed May 29, 2018.

[29] Medicaid Advisory Committee, Maryland Medicaid. Opioid prescribing guidance & policy. https://mmcp.health.maryland.gov/Documents/MMAC/2017/April/MMAC%20Drug%20Utilization%20Review%20Presenttion%20Apr%2017.pdf. Published April 27, 2017. Accessed May 29, 2018.

[30] IQVIA Institute for Human Data Science. Medicine use and spending in the U.S: a review of 2017 and outlook to 2022. https://www.iqvia.com/institute/reports/medicine-use-and-spending-in-the-us-review-of-2017-outlook-to-2022. Accessed May 29, 2018.

[31] Meara E, Horwitz JR, Powell W, State legal restrictions and prescription-opioid use among disabled adults. N Engl J Med. 2016;375(1):44-53. doi:10.1056/NEJMsa1514387 27332619 PMC4985562

[32] Sacarny A, Yokum D, Finkelstein A, Agrawal S Medicare letters to curb overprescribing of controlled substances had no detectable effect on providers. Health Aff (Millwood). 2016;35(3):471-479. doi:10.1377/hlthaff.2015.1025 26953302

[33] Barnett ML, Gray J, Zink A, Jena AB Coupling policymaking with evaluation—the case of the opioid crisis. N Engl J Med. 2017;377(24):2306-2309. doi:10.1056/NEJMp1710014 29236636

[34] von Gunten CF The pendulum swings for opioid prescribing. J Palliat Med. 2016;19(4):348. doi:10.1089/jpm.2016.0079 26905097

[35] Glod SA The other victims of the opioid epidemic. N Engl J Med. 2017;376(22):2101-2102. doi:10.1056/NEJMp1702188 28564563

[36] Feinberg AE, Chesney TR, Srikandarajah S, Acuna SA, McLeod RS; Best Practice in Surgery Group Opioid use after discharge in postoperative patients: a systematic review. Ann Surg. 2018;267(6):1056-1062. doi:10.1097/SLA.0000000000002591 29215370

[37] Hill MV, Stucke RS, Billmeier SE, Kelly JL, Barth RJ Jr Guideline for discharge opioid prescriptions after inpatient general surgical procedures. J Am Coll Surg. 2018;226(6):996-1003. doi:10.1016/j.jamcollsurg.2017.10.012 29198638

[38] Center for Opioid Research and Education Post-surgical opioid guidelines. https://www.solvethecrisis.org/best-practices. Accessed May 30, 2018.

