# Supplementary material for: Assessment of Opioid Prescribing Practices Before and After Implementation of a Health System Intervention to Reduce Opioid Overprescribing
Source: JAMA Netw Open. 2018 Sep 28;1(5):e182908. doi: 10.1001/jamanetworkopen.2018.2908 (PMC6324493; doi:10.1001/jamanetworkopen.2018.2908)
Supplement: Supplement. — eTable 1. Themes of Academic Detailing eTable 2. Initiation and Maintenance of Interventions eFigure. Patient Satisfaction Graph [file jamanetwopen-1-e182908-s001.pdf]

Supplementary Online Content

Meisenberg BR, Grover J, Campbell C, Korpon D. Assessment of Opioid Prescribing Practices Before and After Implementation of a Health System Intervention to Reduce Opioid Overprescribing. *JAMA Netw Open.* 2018;1(5):e182908. doi:10.1001/jamanetworkopen.2018.2908

eTable 1. Themes of Academic Detailing

eTable 2. Initiation and Maintenance of Interventions

eFigure. Patient Satisfaction Graph

This supplementary material has been provided by the authors to give readers additional information about their work.

© 2018 Meisenberg BR et al. *JAMA Network Open.*

eTable 1. Themes of Academic Detailing

**Themes**	**Comment**
Epidemiology of opioid epidemic.	Growth in overdose deaths over time and type of opioid responsible.
Growth of opioid prescribing over past three decades.	Multiple reasons for growth of opioid prescribing; comparison of opioid volumes with similar nations.
Link between clinician prescribing and heroin/fentanyl users.	Most heroin users begin with prescription opioids given or diverted from friends, families. Small, but significant opioid naïve patients will become long term users when exposed.
Phenomenon of opioid diversion.	Survey of opioid overdose patients indicates high level of diversion fueling heroin habit and overdoses.
How prescribing patterns influence likelihood of chronic use. Why this is a prescribing problem and not just a criminal justice problem.	Data showing the impact of “high prescribers” in emergency room and primary care. Link between volume of drugs prescribed within first three months and likelihood of long term use.
Phenomenon of prescribing mismatch between what is prescribed and what patient actually consumes.	Date from post-surgery studies of prescribing and patient intake from surveys shows overprescribing is common.
Guidelines from CDC and others indicating least amount of opioid is best practice.	Habit of over-prescribing for patient convenience and to avoid return visits can no longer be accepted.
Patient satisfaction need not fall with constrained opioid prescribing.	Published data show that lower volumes of opioid at discharge are not associated with less patient satisfaction.
Internal data showing high and unexplained variability in prescribing practices; examples of overt cases of overprescribing.	Discuss monitoring capability and institutional commitment to review practices as a quality measure.

© 2018 Meisenberg BR et al. *JAMA Network Open.*

Desire not to injure patients with acute or chronic pain by depriving them of needed medications.	
EMR based patient education tools:<br>-facilitate discussion of alternatives.<br>-encourage safe storage and disposal	Handout of patient education tools including realistic advice on pain expectation after surgery.<br>EMR tools built to encourage safe use, storage and disposal including listings of drop off sites for unused medications
Other EMR Tools:<br>Facilitated display of inpatient opioid use to right-size discharge prescriptions<br><br>Enhanced referral to substance abuse or mental health counseling.	Reduces discharged overprescribing post operatively and individualizes amount based on actual patient use.<br>Facilitated referral tool built within EMR

© 2018 Meisenberg BR et al. *JAMA Network Open.*

eTable 2. Initiation and maintenance of interventions

Initiative	Time course of initiation
**Physician education (see table 1 for topics)**	Months 1-3; update with medical journal circulation of relevant content on over prescribing
**Creation of opioid prescribing report from electronic medical record with individual and department attribution**	Months 1-3; continuously updated
**Patient/public awareness and education on opioid safety, alternatives to opioids, reasonable pain goals after surgery.**	Month 1 and ongoing with printed after-visit summary patient education
**Electronic medical record facilitate referral to substance abuse coordinator and detoxification services**	Month 2 and ongoing awareness raising
**Creation of accessible 24 hour opioid use tracker to right size discharge opioid prescriptions**	Month 1 and ongoing awareness raising about this functionaltiy
**Physician and administrative leaders given data and responsibility for oversight.**	Month 3 and ongoing
**One on one meetings with outliers**	Month 3 and ongoing
**Sharing published literature on over prescribing with prescribers**	Continuously distributed to medical staff
**Creation of multi-disciplinary opioid task force**	Month 3 and ongoing
**Creation of standard prescribing practices by individual departments or groups of surgeons**	Month 3 and ongoing

© 2018 Meisenberg BR et al. *JAMA Network Open.*

eFigure. Patient Satisfaction Graph

Emergency Department- Pain Control

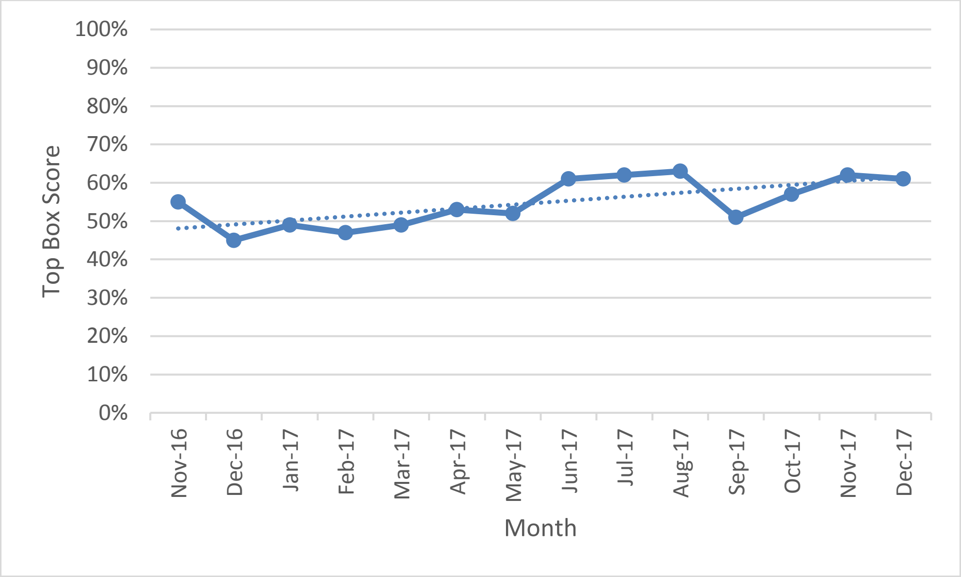

© 2018 Meisenberg BR et al. *JAMA Network Open.*
